# Comparative plastome analysis and phylogenetic relationships of Chinese *Myricaria* species (Tamaricaceae)

**DOI:** 10.3389/fpls.2026.1926827

**Published:** 2026-08-04

**Authors:** Haiwen Li, Yiheng Wang, Yanlei Liu, Shuo Shi

**Affiliations:** 1 College of Life Sciences and Technology, Tarim University, Alar, China; 2 State Key Laboratory for Quality Ensurance and Sustainable Use of Dao-di Herbs, National Resource Center for Chinese Materia Medica, Academy of Chinese Medical Sciences, Beijing, China; 3 School of Landscape and Ecological Engineering, Hebei University of Engineering, Handan, China; 4 College of Life Science, Hebei Normal University, Shijiazhuang, China

**Keywords:** medicinal utilization, Myricaria, phylogeny, plastome, species identification

## Abstract

The genus *Myricaria* (Tamaricaceae) comprises shrubs widely distributed across arid and high-altitude regions of Eurasia, with China representing an important diversity center, particularly in the Qinghai–Tibetan Plateau and adjacent areas. To investigate plastome evolution and phylogenetic relationships within this genus, we sequenced and comparatively analyzed complete plastomes of five *Myricaria* species from China, together with those of *Reaumuria* as an outgroup. All plastomes exhibited a typical quadripartite structure and highly conserved gene composition, containing 127–135 genes, indicating strong structural stability across the genus. Despite the overall conservation of plastome architecture, minor size differences were observed and were mainly associated with contraction and expansion of inverted repeat (IR) boundaries. Comparative analyses revealed heterogeneous patterns of sequence divergence, with nucleotide variation primarily concentrated in intergenic regions and a limited number of protein-coding genes, whereas IR regions showed relatively high conservation. Simple sequence repeats (SSRs) and long repeats displayed species-specific distribution patterns, contributing to localized plastome variation. Sliding window analysis identified several mutation hotspots mainly located in single-copy regions, suggesting their potential utility for species identification and phylogenetic studies. Phylogenomic analyses based on complete plastome sequences strongly supported the monophyly of *Myricaria* and resolved major relationships among sampled species. However, limited resolution among some closely related taxa suggests recent diversification and possible incomplete lineage sorting. Overall, *Myricaria* plastomes show a pattern of highly conserved genome structure coupled with localized sequence divergence, providing valuable resources for phylogenetic resolution, species identification, and plastome evolutionary studies.

## 1 Introduction

*Myricaria* Desv. (Tamaricaceae) is a genus of deciduous shrubs widely distributed in arid, semi-arid, and high-altitude regions of Eurasia, where it plays important ecological and systematic roles in dryland vegetation (Editorial Committee of the Flora of China CAoS, 1990; Wang et al., 2009). The genus comprises approximately 11–13 species worldwide, with China representing its modern distribution center and diversity hotspot, harboring 10 species and one variety. These taxa are mainly distributed across the Qinghai–Tibetan Plateau and adjacent regions, where they inhabit alpine valleys, gravel riverbeds, and arid basins. Adaptation to these harsh environments has resulted in traits such as drought tolerance, cold resistance, salt tolerance, and strong sand-fixation ability, making the species ecologically important for soil conservation, vegetation restoration, and the maintenance of fragile alpine ecosystems (Editorial Committee of the Flora of China CAoS, 1990; Wang et al., 2009; Zhang et al., 2014; Hu et al., 2023; Makowska-Was̨ et al., 2026). In addition, several species possess potential medicinal and ecological restoration value.

Species of *Myricaria* are morphologically similar, and species delimitation has traditionally relied on a limited number of diagnostic characters, including inflorescence architecture, bract morphology, and fruit characteristics. However, these characters often show considerable variation and overlap among species, making accurate identification and taxonomic classification challenging. Although several molecular phylogenetic studies based on nuclear ITS (internal transcribed spacer) and a few plastid DNA fragments have provided preliminary insights into relationships within the genus, the available genomic resources remain extremely limited, particularly for Chinese species (Wang et al., 2009). Consequently, phylogenetic relationships among several closely related taxa remain insufficiently resolved, highlighting the need for more comprehensive genomic data.

Plastid genomes (plastomes) have become valuable resources for plant evolutionary and systematic studies because of their highly conserved structure, predominantly uniparental inheritance, low recombination rate, and moderate sequence variation (Yan et al., 2024; Hu et al., 2023; Chen et al., 2024; Lubna et al., 2024; Sun et al., 2024). Compared with traditional molecular markers, complete plastome sequences provide substantially greater phylogenetic information and enable comprehensive comparative analyses of genome organization, repeat sequences, simple sequence repeats (SSRs), codon usage, and variation in inverted repeat (IR) boundaries. These characteristics make plastome data particularly useful for species identification, comparative genomics, DNA barcode development, and phylogenetic reconstruction among closely related taxa (Li et al., 2015; Zhang et al., 2021; Wang et al., 2025; Liu et al., 2025c; Liu et al., 2025b; Liu et al., 2025a; Shen et al., 2025; Wang et al., 2026a; Wang et al., 2026b). Despite these advantages, plastome resources for *Myricaria* remain scarce. To date, complete plastome sequences have been reported for only a few species, including *Myricaria prostrata*, *M. laxiflora*, and *M. elegans* (Hu et al., 2023), leaving most Chinese species without genomic resources. This limited taxonomic sampling has hindered comprehensive comparative analyses of plastome evolution and restricted robust phylogenetic inference within the genus.

In this study, we generated and analyzed complete plastome sequences representing Chinese *Myricaria* species to establish a more comprehensive genomic dataset for this genus. We characterized plastome structural features, gene content, codon usage bias, repeat sequence composition, SSR distribution, and IR boundary variation, and identified highly variable regions with potential utility as molecular markers. Furthermore, we reconstructed phylogenetic relationships using complete plastome sequences to clarify interspecific relationshipswa within *Myricaria*. Our study provides valuable genomic resources for this taxonomically important genus and contributes to future studies of plastome evolution, species identification, and phylogenetic relationships in *Myricaria*.

## 2 Materials and methods

### 2.1 Plant materials and DNA sampling

Fresh young leaves of six samples representing three *Myricaria* species [*Myricaria paniculata* (2), *Myricaria wardii* (2), and *Myricaria pulcherrima* (1)] and one *Reaumuria* species (*Reaumuria kaschgarica*) were collected from natural populations across the Qinghai-Tibetan Plateau and adjacent regions in China (**Table 1**). All sampled individuals were identified based on morphological characteristics following the Flora of China. Voucher specimens were deposited in the Herbarium of Tarim University. Healthy leaf tissues were immediately dried in silica gel for subsequent DNA extraction. In addition, 10 complete plastome sequences representing six *Myricaria* species and three plastome sequences representing two *Reaumuria* species were retrieved from the NCBI public database. Detailed information for all samples and plastome sequences is provided in **Table 1**.

**Table 1:** 

Species Name	Accession No.	Sample Name	Herbarium No.	Total (bp)	LSC (bp)	IRa (bp)	SSC	PCG	rRNA	tRNA
*Myricaria paniculata*	C_AA120038.1	MPA2	HTU00021	154700	84493	25953	18301	83	8	36
*Myricaria paniculata*	C_AA120037.1	MPA1	HTU00020	154703	84441	26006	18250	83	8	36
*Myricaria elegans*	MZ489116	MZ489116	Public Data	155245	84846	26054	18291	83	8	36
*Reaumuria songarica*	MW760848	MW760848	Public Data	157072	86544	26044	18440	83	8	37
*Myricaria prostrata*	MN088847	MN088847	Public Data	155230	84425	26562	17681	84	8	37
*Reaumuria kashgarica*	C_AA120041.1	RKA	HTU00059	155597	85899	26068	17562	87	8	36
*Reaumuria trigyna*	MK397893	MK397893	Public Data	154533	84812	26057	17607	85	8	37
*Myricaria laxiflora*	MN867948	MN867948	Public Data	154971	84709	26004	18254	85	8	37
*Myricaria wardii*	C_AA120039.1	81	HTU00048	154710	84442	26009	18250	86	8	37
*Myricaria wardii*	C_AA120040.1	82	HTU00052	154750	84480	26009	18252	86	8	37
*Myricaria pulcherrima*	C_AA120036.1	83	HTU00040	154764	84431	26010	18313	86	8	37
*Reaumuria songarica*	OM112328	OM112328	Public Data	154699	85095	26032	17540	87	8	37
*Myricaria laxiflora*	OP778385	OP778385	Public Data	154897	84728	25966	18237	87	8	37
*Myricaria bracteata*	PP072247	PP072247	Public Data	154709	84441	26009	18250	89	8	37
*Myricaria squamosa*	OP778391	OP778391	Public Data	154691	84385	26001	18304	89	8	38
*Myricaria squamosa*	OP778392	OP778392	Public Data	154695	84388	26001	18305	89	8	38
*Myricaria rosea*	OP778411	OP778411	Public Data	155105	84540	26140	18285	89	8	38
*Myricaria elegans*	OP778415	OP778415	Public Data	155265	84825	26073	18294	89	8	38
*Myricaria rosea*	OP778410	OP778410	Public Data	155300	84748	26134	18284	89	8	38

### 2.2 DNA extraction, sequencing, and plastome assembly

Total genomic DNA was extracted using a modified CTAB method (Li et al., 2013). DNA quality and concentration were assessed using 1.5% agarose gel electrophoresis and a NanoDrop spectrophotometer (Thermo Fisher Scientific, USA). High-quality DNA samples were used to construct paired-end libraries with an insert size of approximately 350 bp and sequenced on the Illumina HiSeq X Ten platform, generating 150 bp paired-end reads. Raw sequencing reads were filtered to remove low-quality reads, adapter sequences, and ambiguous bases using NGS QC Toolkit v2.3 (Patel and Jain, 2012). Clean reads were then used for *de novo* assembly of plastomes using GetOrganelle v1.7.7.0, with default parameters (-F embplant_pt -R 15 -k 21, 45, 65, 85, 105) optimized for plant plastome assembly (Jin et al., 2020). Assembly graphs were visually inspected and refined when necessary. The complete circular plastomes were validated by read mapping using Geneious Prime 2021.2.2.

### 2.3 Plastome annotation and visualization

The assembled plastomes were annotated using GeSeq (https://chlorobox.mpimp-golm.mpg.de/geseq.html) and further manually corrected by comparison with the previously published *Myricaria bracteata* (PP072247) plastome (Tillich et al., 2017). Protein-coding genes, tRNA genes, and rRNA genes were identified based on reference annotation. Gene boundaries and intron-exon structures were verified in Geneious Prime 2021.2.2. Circular plastome maps were generated using Plastidhub (https://www.plastidhub.cn/). Codon usage and relative synonymous codon usage (RSCU) were calculated using CodonW v1.4.4 (https://codonw.sourceforge.net/). GC content and genome structural features were also analyzed.

### 2.4 Comparative plastome analysis

Genome alignment was performed using MAFFT v7.526 with default settings. Expansion and contraction of inverted repeat (IR) regions were analyzed using IRscope (https://irscope.shinyapps.io/irapp/) (Katoh and Standley, 2013; Amiryousefi et al., 2018). Boundaries between LSC, SSC, and IR regions were compared across species to detect structural variation. Simple sequence repeats (SSRs) were identified using MISA-web (Talavera and Castresana, 2007). The minimum repeat thresholds were set to 10 for mononucleotide repeats, 5 for dinucleotide repeats, 4 for trinucleotide repeats, and 3 for tetra-, penta-, and hexanucleotide repeats. Long repeats, including forward, reverse, palindromic, and complementary repeats, were detected using REPuter with a minimum repeat size of 30 bp, a Hamming distance of 3, and a sequence identity of ≥90% (Kurtz et al., 2001). Nucleotide diversity (Pi) was calculated using DnaSP v6 to identify highly variable regions among plastomes (Julio et al., 2017). Sliding window analysis was performed with a step size of 200 bp and a window length of 600 bp. Regions with high Pi (π) values were considered potential mutation hotspots and candidate DNA barcodes. To evaluate the species discrimination power of the potential candidate DNA barcode regions, maximum likelihood (ML) phylogenetic trees were reconstructed for each highly variable locus. The phylogenetic analyses were performed using the same procedures described in Section 2.5.

### 2.5 Phylogenomic analysis

Complete plastome sequences of *Myricaria* species and related taxa including *Reaumuria songarica*, *R. kaschgarica* and *R. trigyna* as outgroups were aligned using MAFFT v7.526 with the --auto strategy. Poorly aligned regions were removed using Gblocks v0.91b to improve alignment quality. Maximum likelihood (ML) phylogenetic analysis was conducted using IQ-TREE v2.4.0 with the best-fit substitution model selected by ModelFinder (Kalyaanamoorthy et al., 2017; Minh et al., 2020). Node support was assessed using 1, 000 ultrafast bootstrap (UFBoot) replicates and the SH-aLRT test with 1, 000 replicates. Bayesian inference (BI) phylogenetic analyses were conducted using MrBayes v3.2.7a (Ronquist et al., 2012). The analysis was run with four independent Markov chain Monte Carlo (MCMC) chains for 100, 000, 000 generations, sampling every 1, 000 generations. The initial 25% of sampled trees were discarded as burn-in. The remaining trees were used to construct a majority-rule consensus tree, and posterior probabilities were used to assess branch support. The resulting phylogenetic tree was visualized and edited using FigTree v1.4.4 (https://tree.bio.ed.ac.uk/software/figtree/).

## 3 Results

### 3.1 Plastomes genome information of *Myricaria* and *Reaumuria*

**Figure 1:**
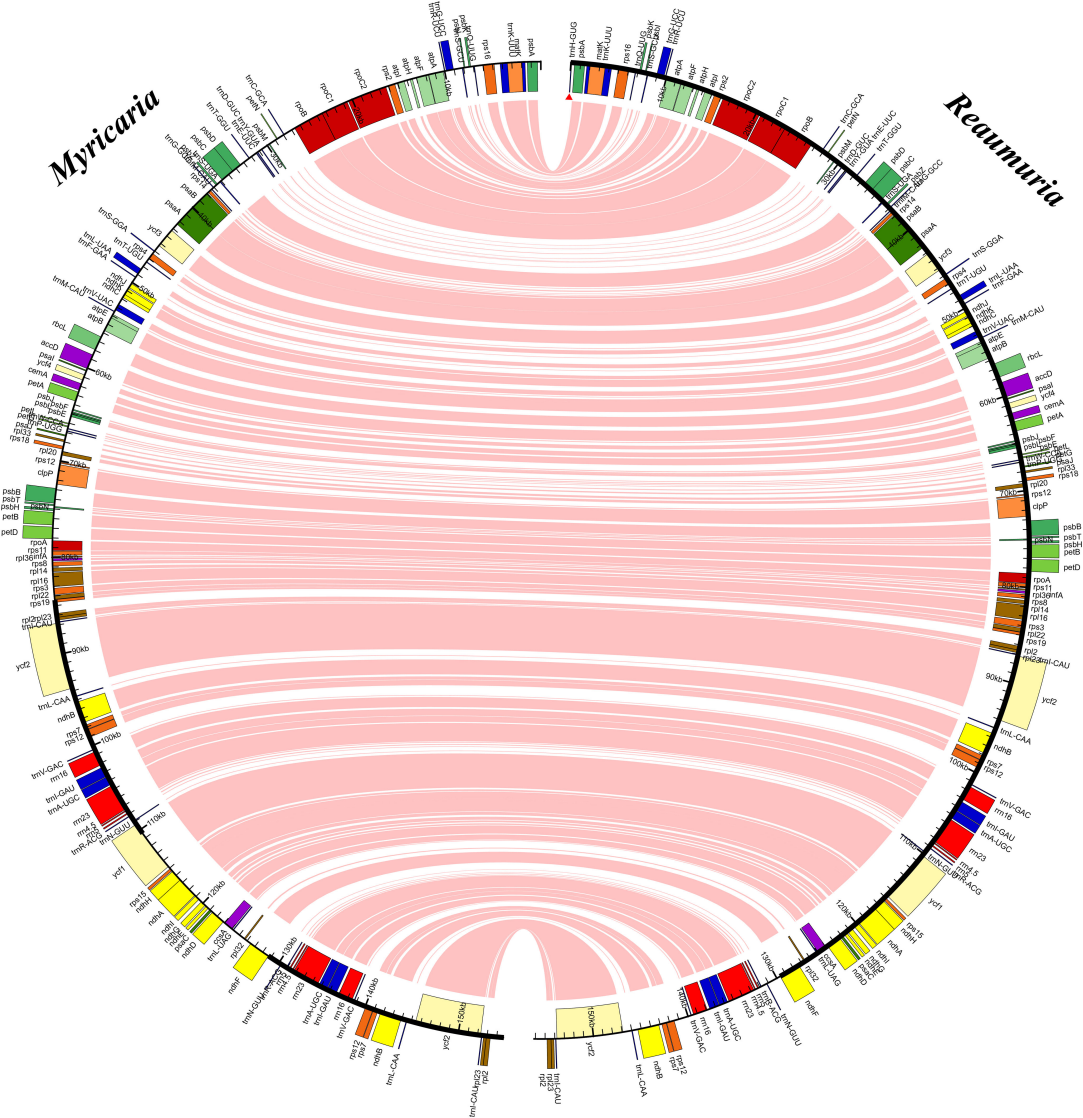
Physical maps of the plastomes of *Myricaria* and *Reaumuria.* Protein-coding genes, tRNA genes, and rRNA genes are color-coded according to their functional categories. The large single-copy (LSC), small single-copy (SSC), and inverted repeat (IR) regions are indicated on the maps. Pink ribbons connect homologous genomic regions shared between the two plastomes, illustrating the high level of structural conservation and collinearity.

All *Myricaria* plastomes obtained in this study displayed a conserved circular configuration, with a typical quadripartite structure consisting of a large single-copy (LSC) region, a small single-copy (SSC) region, and two inverted repeat (IR) regions (**Table 1**). Genome lengths were not uniform across taxa, ranging from 154, 691 bp (*Myricaria squamosa*) to 155, 300 bp (*Myricaria rosea*), indicating only slight size variation among species (**Table 1**). While the LSC, SSC, and IR regions of *Myricaria* ranged from 84, 385–84, 846 bp, 17, 681–18, 313 bp, and 25, 953–26, 562 bp, respectively. While The LSC, SSC, and IR regions of *Reaumuria* ranged from 84, 812–86, 544 bp, 17, 540–18, 440 bp, and 26, 032–26, 068 bp, respectively (**Table 1**). GC content was highly similar among species (36.4–36.6%), with the IR regions consistently exhibiting a higher GC content than the LSC and SSC regions. Gene order and genome organization were highly conserved across all *Myricaria* species and the outgroup *Reaumuria* species (**Figure 1**).

Across all analyzed plastomes, 127–135 genes were identified, including 83–89 protein-coding genes (PCGs), 36–38 tRNAs, and 8 rRNAs (**Table 1**). Gene order was highly similar among all species. Differences in gene number were mainly associated with duplicated genes located in the IR regions and annotation of several genes near IR boundaries. Genes such as *ycf1*, *rps12*, and *clpP* showed differences in gene length or exon structure among species.

### 3.2 Codon usage analysis

**Figure 2:**
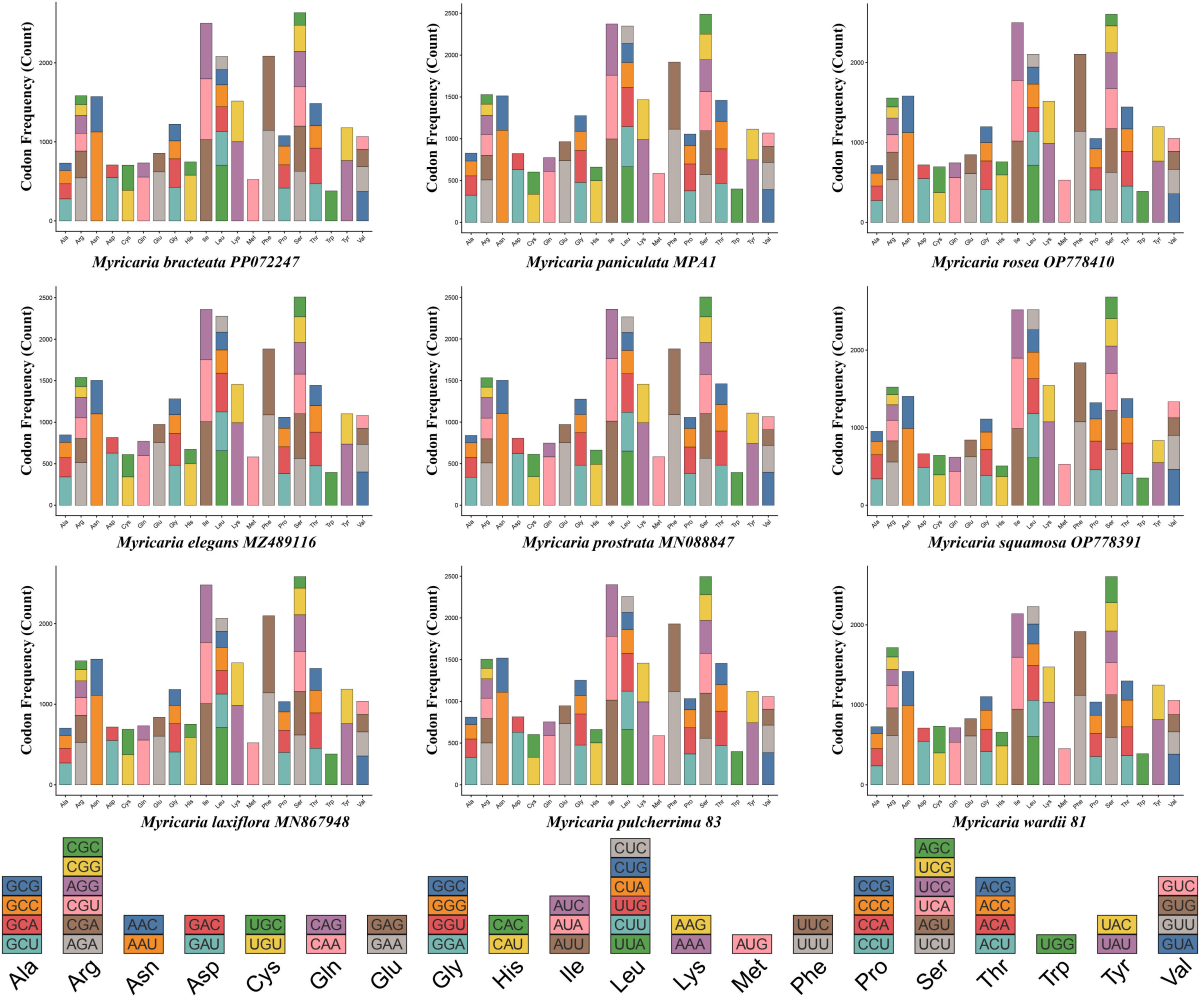
Codon usage bias analysis of *Myricaria* species. Relative synonymous codon usage (RSCU) values of the 61 synonymous codons in the plastomes of different *Myricaria* species. Each panel represents one species, and the height of each bar indicates the RSCU value of the corresponding codon. Amino acids are indicated below the corresponding codons.

Codon usage patterns were highly consistent across all *Myricaria* species. A total of 61 synonymous codons encoding 20 amino acids were identified in the protein-coding genes. The total codon set derived from protein-coding sequences revealed a clear preference toward A/U-ending codons, a pattern commonly associated with plastid genome evolution in angiosperms. Among amino acids, leucine was the most frequently represented [7.81% (*Myricaria bracteata* PP072247) – 9.74% (*Myricaria squamosa* OP778392) of total codons], whereas cysteine remained the least abundant [2.30% (*Myricaria paniculata* MPA1) – 2.82% (*Myricaria wardii* 81) of total codons]. Overall, codon usage bias appeared evolutionarily constrained rather than species-specific and 31–33 codons exhibited RSCU values greater than 1.0 (**Figure 2**).

### 3.3 Repeat sequence analysis

**Figure 3:**
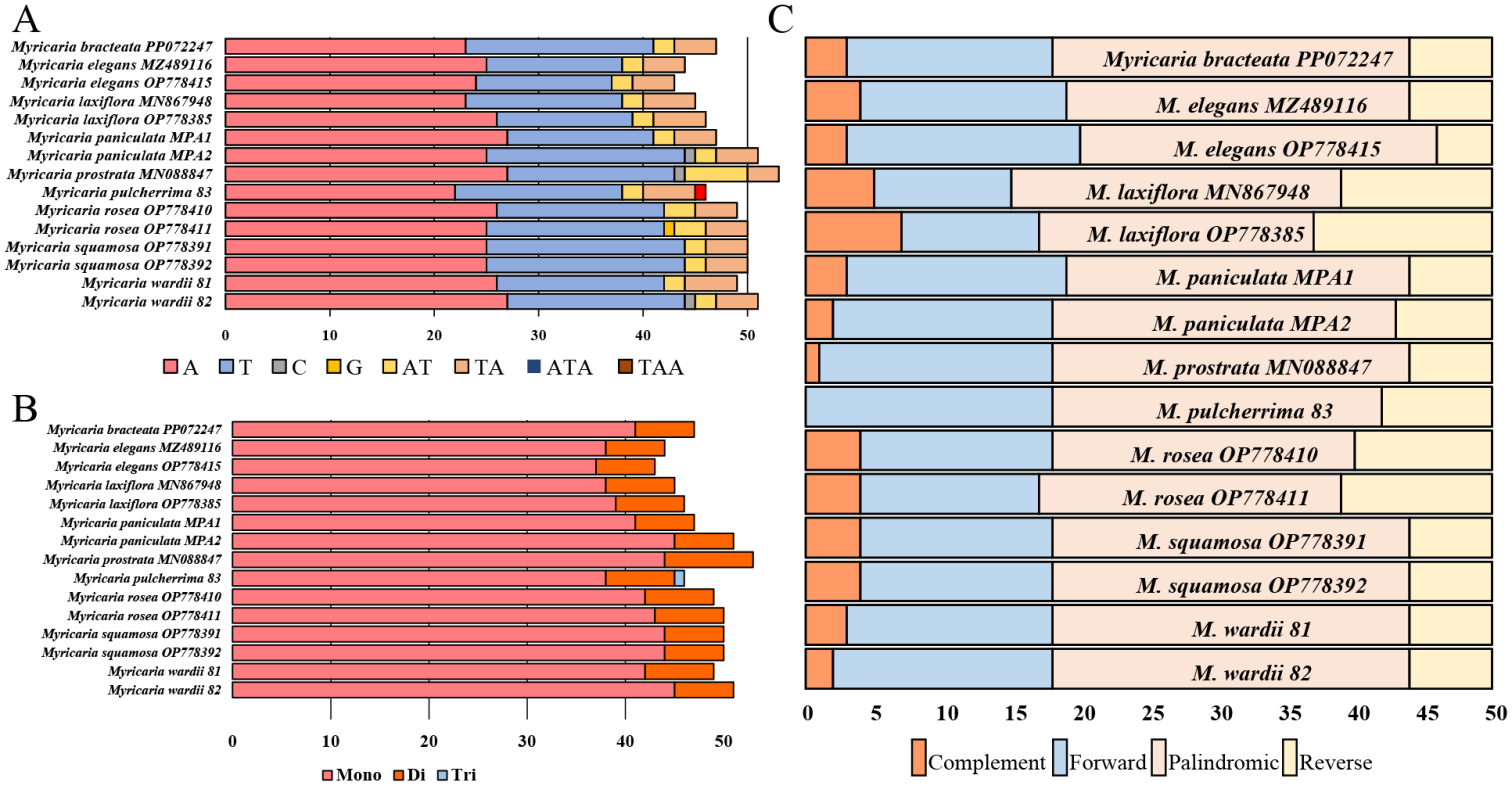
Distribution of repetitive sequences in the plastomes of *Myricaria* species. **(A)** Distribution by individual SSR motif types among different samples; **(B)** Distribution by major SSR categories among different samples; **(C)** Summary of large repeat types in the plastomes of *Myricaria* species.

Although plastome sequences were highly conserved at the structural level, repeat-related elements introduced measurable genomic heterogeneity. SSR loci were unevenly distributed among species, with counts ranging from 44 to 53. Mononucleotide repeats dominated the SSR landscape (mostly A/T types, more than 90%), while more complex repeat motifs occurred less frequently but contributed disproportionately to sequence variability (**Figures 3A, B**). Long repeat structures (forward, palindromic, reverse, and complementary repeats) showed a similar pattern: they were rare in coding regions but enriched in intergenic spacers. This distribution suggests that non-coding regions serve as primary reservoirs of microstructural variation in *Myricaria* plastomes. Overall, palindromic repeats were the most abundant (20-26), followed by forward (10-13), reverse (3-12), and complementary (1-6) repeats (**Figure 3C**).

### 3.4 Identification of highly variable regions

**Figure 4:**
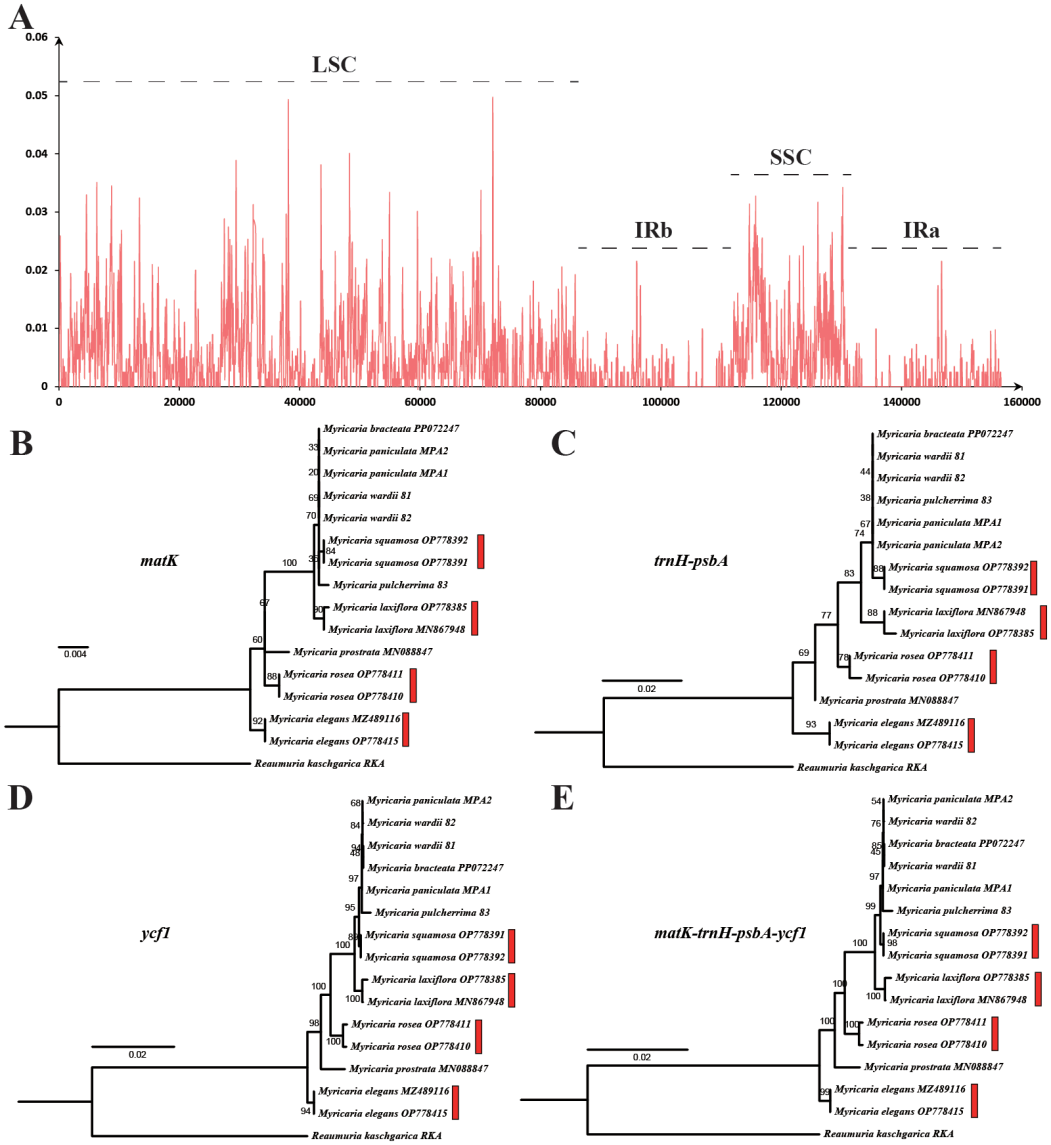
Nucleotide diversity and phylogenetic analyses based on highly variable plastome regions of *Myricaria*. **(A)** Nucleotide diversity (Pi) across the plastomes of *Myricaria* species. **(B–E)** Maximum likelihood (ML) phylogenetic trees based on *matK* **(B)**, *trnH–psbA* **(C)**, *ycf1* **(D)**, and the combined dataset of the three highly variable regions **(E)**. Numbers at the nodes indicate bootstrap support values.

Despite the overall conservation of plastome sequences, comparative analysis revealed that divergence was not evenly distributed across the genome (**Figure 4A**). Instead, variability was concentrated in discrete regions, particularly intergenic spacers and a few fast-evolving coding genes. Sliding window analysis identified several peaks of nucleotide diversity (Pi = 0-0.05), forming clear mutation hotspots. Most coding regions exhibited Pi values below 0.01, whereas several intergenic regions showed markedly higher nucleotide diversity. The highest Pi values (0.05) were detected at 38, 000 and 90, 000 bp in the LSC region (highly variable regions including *matK*, *ycf1*, and *trnH-psbA*). These regions were consistently located in non-coding segments, while most coding regions remained highly conserved. The uneven distribution of variability suggests that evolutionary constraints operate strongly on functional genes, whereas neutral or nearly neutral regions accumulate most of the detectable divergence. The SSC region exhibited an intermediate level of nucleotide diversity between the LSC and IR regions. In addition, regions with relatively high nucleotide diversity were predominantly distributed in the two single-copy regions, whereas the two IR regions exhibited strong conservation in nucleotide diversity (**Figure 4A**). All three potential hypervariable regions, as well as their combined dataset, successfully discriminated the four *Myricaria* species (**Figures 4B–E**).

### 3.5 IR boundary comparison

**Figure 5:**
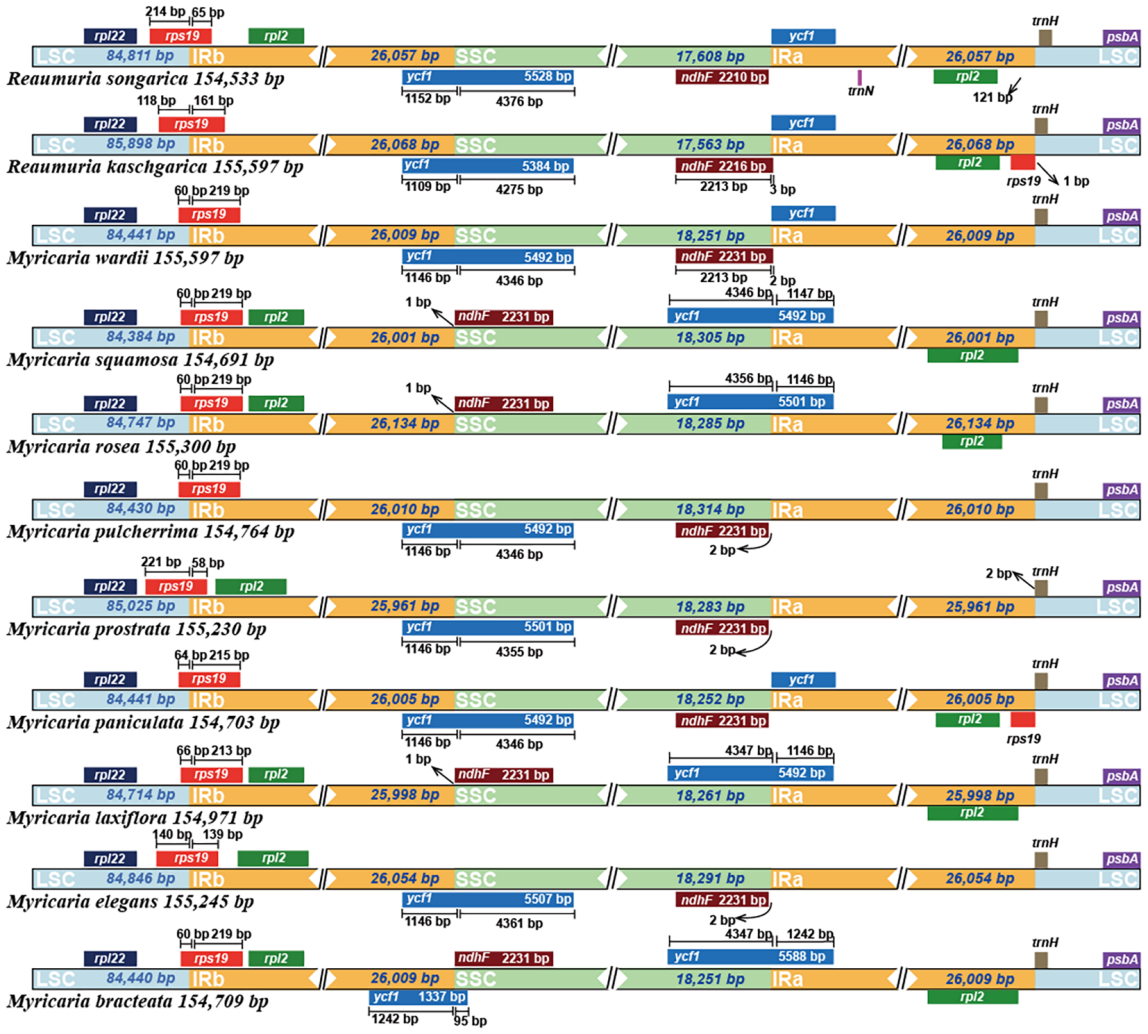
Expansion and contraction of junctions among different regions in the plastomes of *Myricaria* species. The positions of genes located near the four junctions (LSC/IRb, IRb/SSC, SSC/IRa, and IRa/LSC) are shown for each plastome. Colored boxes represent annotated genes, and the numbers indicate the lengths of the corresponding genomic regions or the distances between genes and junctions (bp).

Comparison of the junctions between the inverted repeat (IR) and single-copy (SC) regions revealed a highly similar boundary organization among the analyzed *Myricaria* plastomes (**Figure 5**). The overall arrangement of genes flanking the four junctions (LSC/IRb, IRb/SSC, SSC/IRa, and IRa/LSC) was conserved across all species. At the LSC/IRb boundary, the *rps19* gene consistently spanned the junction, with a portion of the gene extending into the IRb region, while the adjacent *rpl2* gene was entirely located within the IR region. At the IRb/SSC junction, the *ycf1* gene crossed the boundary in all plastomes, with 1, 337-5, 588 bp located within the SSC region. The neighboring *ndhF* gene was positioned entirely within the SSC region, and the distance between *ycf1* and *ndhF* ranged from 5, 284 to 5, 528 bp among species (**Figure 5**). At the SSC/IRa boundary, the *ycf1* gene extended into the IRa region, whereas *ndhF* remained completely within the SSC region. The overlap between *ycf1* and the IRa region showed only slight variation among species. At the IRa/LSC junction, the *rpl2* gene was consistently located within the IRa region, whereas *psbA* was entirely positioned in the LSC region. The distance between the IRa/LSC junction and *psbA* varied only slightly among plastomes.

Overall, only minor shifts were detected in the positions of the boundary-associated genes (*rps19*, *ycf1*, *ndhF*, *rpl2*, and *psbA*) among the analyzed plastomes. No gene gain, gene loss, or large-scale expansion or contraction of the IR regions was observed, and the overall boundary organization remained highly conserved across all *Myricaria* species (**Figure 5**).

### 3.6 Phylogenetic relationships

**Figure 6:**
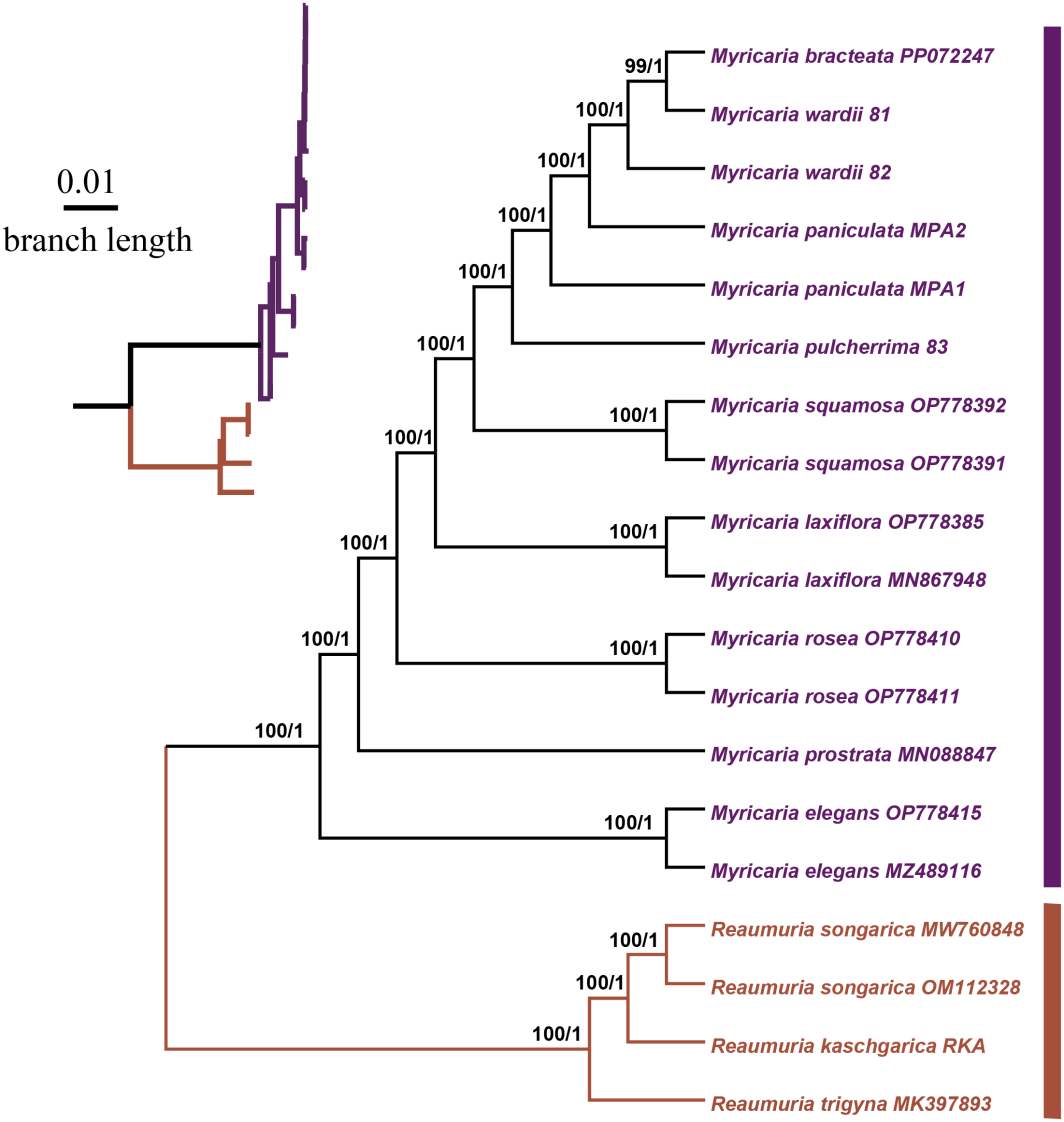
Maximum likelihood (ML) and Bayesian inference (BI) phylogenetic relationships of *Myricaria* species based on complete plastomes. Numbers at the nodes indicate ML bootstrap support (BS) values and Bayesian posterior probabilities (PP).

Phylogenetic analyses based on complete plastome sequences produced identical topologies using both maximum likelihood (ML) and Bayesian inference (BI) methods, indicating the robustness of the inferred relationships (**Figure 6**). All major nodes received strong statistical support, with high ML bootstrap values and BI posterior probabilities. All *Myricaria* accessions formed a well-supported monophyletic clade, which was clearly separated from the outgroup genus *Reaumuria*. Within *Reaumuria*, the two accessions of *R. songarica* clustered together with strong support and were sister to *R. kaschgarica*, whereas *R. trigyna* occupied the basal position of the outgroup clade (**Figure 6**). Within *Myricaria*, accessions belonging to the same species consistently clustered together. The two accessions of *M. wardii*, *M. paniculata*, *M. squamosa*, *M. laxiflora*, *M. rosea*, and *M. elegans* each formed well-supported intraspecific clades, indicating high consistency between independently sequenced plastomes. *Myricaria prostrata* occupied an independent branch, whereas *M. bracteata* was recovered as sister to the clade containing *M. wardii*, *M. paniculata*, and *M. pulcherrima*. These species together formed a distinct lineage within the genus. Although the overall topology was well resolved, branch lengths varied among lineages. The terminal branches of *M. bracteata*, *M. wardii*, *M. paniculata*, and *M. pulcherrima* were relatively short compared with those of several other species, whereas deeper nodes separating the major species groups were supported by longer internal branches. Overall, the complete plastome dataset provided sufficient phylogenetic signal to resolve relationships among the sampled *Myricaria* species with high confidence.

## 4 Discussion

### 4.1 Comparative plastome analyses reveal a highly conserved plastome organization in *Myricaria*

Comparative analyses of the plastomes demonstrated that all *Myricaria* species shared a typical quadripartite structure consisting of a large single-copy (LSC) region, a small single-copy (SSC) region, and two inverted repeat (IR) regions. Genome size, GC content, gene order, and gene composition were highly conserved across all sampled species, with plastome lengths varying only from 154, 691 to 155, 300 bp. Similar levels of structural conservation have also been reported in other members of Tamaricaceae, including *Reaumuria* and *Tamarix*, as well as in numerous angiosperm lineages, indicating that plastome organization is generally stable within closely related taxa (Hughes and Atchison, 2025; Mower and Vickrey, 2018; Zhao et al., 2020; Hu et al., 2023; Liu et al., 2024; Li et al., 2026).

The slight variation in plastome size observed among *Myricaria* species was mainly associated with small differences at the IR/SC boundaries rather than large structural rearrangements or gene gain and loss. Genes located near the junctions, including *rps19*, *ycf1*, *ndhF*, and *rpl2*, showed only minor positional shifts among species, whereas overall genome organization remained unchanged. Similar IR boundary variation has been widely reported in angiosperm plastomes and is generally considered one of the primary contributors to small differences in plastome size among closely related species (Liu et al., 2025c; Li et al., 2026).

The high degree of structural conservation observed in *Myricaria* is consistent with the generally conserved nature of plastid genomes, which typically exthibit stable genome organization, conserved gene content, and limited structural variation during evolution (Dong et al., 2015; Mower and Vickrey, 2018). Collectively, these results indicate that plastome architecture is highly conserved within *Myricaria*, providing a reliable genomic framewortk for comparative genomic and phylogenetic studies.

### 4.2 Sequence variation provides useful molecular resources for *Myricaria*

Although plastome structure was highly conserved, comparative analyses detected measurable sequence variation among *Myricaria* species. SSR analysis showed that mononucleotide repeats, particularly A/T-rich motifs, were the predominant repeat type, whereas dinucleotide and longer repeat motifs occurred much less frequently. Similar SSR composition has been reported in plastomes of Tamaricaceae and many other angiosperm groups, reflecting the AT-rich characteristics of plastid genomes (Yu et al., 2011; Zhang et al., 2021; Li et al., 2026). Long repeat analysis further revealed that most repeats were distributed in intergenic regions, while relatively few occurred within coding sequences, a distribution pattern that is also commonly observed in plastomes of related taxa.

Sliding-window analysis identified several highly variable regions distributed mainly within the LSC and SSC regions, whereas the IR regions remained comparatively conserved. Among these variable loci, coding genes such as *ycf1* and *matK*, together with intergenic spacers including *trnH-psbA*, exhibited relatively high nucleotide diversity. These loci have also been reported as mutation hotspots in numerous plant groups and have been widely used in comparative genomics, phylogenetic reconstruction, and DNA barcode development (Yu et al., 2011; Dong et al., 2015; Mower and Vickrey, 2018; Zhang et al., 2021; Liu et al., 2025c; Li et al., 2026).

Compared with conventional plastid DNA barcodes, complete plastome sequences provide substantially more informative characters for phylogenetic analyses. Nevertheless, the highly variable regions identified in this study may represent practical molecular markers for rapid species identification and future population genetic studies, particularly when whole-plastome sequencing is not feasible (**Figure 4**). Further evaluation using broader taxonomic and population sampling will help determine their effectiveness as candidate DNA barcodes for *Myricaria*.

### 4.3 Plastome phylogeny improves the understanding of relationships within *Myricaria*

Phylogenetic analyses based on complete plastome sequences recovered identical topologies using both maximum likelihood and Bayesian inference methods, with strong statistical support for most nodes. All sampled *Myricaria* accessions formed a well-supported monophyletic clade that was clearly separated from the outgroup genus *Reaumuria*. Species represented by multiple accessions consistently clustered together, indicating good agreement between independently assembled plastomes and supporting the reliability of the plastome dataset for phylogenetic reconstruction. Previous phylogenetic studies of *Myricaria* were primarily based on nuclear ITS sequences and a limited number of plastid loci, which provided only moderate resolution for several interspecific relationships (Wang et al., 2009). In comparison, the complete plastome dataset generated in the present study substantially increased the number of phylogenetically informative characters and improved support for most internal branches. These results demonstrate the value of plastome-scale datasets for resolving relationships among closely related species within the genus.

Although the overall topology was well resolved, several closely related species were separated by relatively short branches, indicating limited plastome sequence divergence among these taxa. Because plastomes represent a single, predominantly maternally inherited genome, plastome phylogenies alone may not fully reflect species evolutionary history (Dong et al., 2025). Therefore, integrating plastome data with evidence from nuclear genomes and broader taxon sampling will provide a more comprehensive understanding of phylogenetic relationships within *Myricaria*.

### 4.4 Limitations and future perspectives

This study provides one of the most comprehensive comparative plastome datasets currently available for *Myricaria*. Nevertheless, several limitations should be acknowledged. First, plastomes represent only a single non-recombining genome and therefore provide information from only one component of the evolutionary history of a species. Second, although most currently recognized Chinese species were included, additional sampling covering broader geographic ranges and intraspecific populations would further improve the understanding of plastome diversity within the genus. Future studies integrating plastome sequences with nuclear genomic data, population-level sampling, and phylogeographic analyses will provide a more comprehensive understanding of species relationships and evolutionary history in *Myricaria*. In addition, the highly variable plastome regions identified in this study should be evaluated across larger sample sizes to determine their potential as efficient DNA barcodes and molecular markers for species identification, population genetics, and conservation studies.

## 5 Conclusions

This study presents a comprehensive comparative plastome analysis of Chinese *Myricaria* species and provides new genomic resources for this taxonomically important genus. Comparative analyses demonstrated that *Myricaria* plastomes are highly conserved in genome size, quadripartite structure, gene content, gene order, and IR boundary organization. Several highly variable regions, including *ycf1*, *matK*, and *trnH–psbA*, were identified and showed potential as candidate DNA barcodes for species discrimination. Phylogenetic analyses based on complete plastome sequences recovered a well-supported monophyletic *Myricaria* clade and improved the resolution of interspecific relationships compared with previous studies using conventional molecular markers. Together, these findings provide valuable genomic resources and a robust phylogenetic framework for future systematic and evolutionary studies of *Myricaria*.

## Data Availability

All plastomes have been uploaded to National Genomics Data Center (NGDC, https://ngdc.cncb.ac.cn/). The accession numbers of the datasets are provided in **Table 1**.

## References

[B1] Amiryousefi A. Hyvönen J. Poczai P. (2018). IRscope: an online program to visualize the junction sites of chloroplast genomes. Bioinformatics 34, 3030–3031. doi: 10.1093/bioinformatics/bty220 29659705

[B2] Chen L.-Q. Li X. Yao X. Li D.-Z. Barrett C. dePamphilis C. . (2024). Variations and reduction of plastome are associated with the evolution of parasitism in Convolvulaceae. Plant Mol. Biol. 114, 40. doi: 10.1007/s11103-024-01440-1 38622367

[B3] Dong W. Xu C. Li C. Sun J. Zuo Y. Shi S. . (2015). ycf1, the most promising plastid DNA barcode of land plants. Sci. Rep. 5, 8348. doi: 10.1038/srep08348 25672218 PMC4325322

[B4] Dong Y. Cao Q. Yu K. Wang Z. Chen S. . (2025). Chloroplast phylogenomics reveals the maternal ancestry of cultivated chrysanthemums. Genomics Commun. 2, e019. doi: 10.48130/gcomm-0025-0019

[B5] Editorial Committee of the Flora of China CAoS (1990). Flora of China, Volume 25, Part 2: Chenopodiaceae (Amaranthaceae) Vol. 25 (Beijing: Science Press).

[B6] Hu H. Wang Q. Hao G. Zhou R. Luo D. Cao K. . (2023). Insights into the phylogenetic relationships and species boundaries of the Myricaria squamosa complex (Tamaricaceae) based on the complete chloroplast genome. PeerJ 11, e16642. doi: 10.7717/peerj.16642 38099308 PMC10720482

[B7] Hughes C. Atchison G. (2015). The ubiquity of alpine plant radiations: from the Andes to the Hengduan Mountains. New Phytol. 207, 275–282. doi: 10.1111/nph.13230 25605002

[B8] Jin J.-J. WenBin Y. Jun-Bo Y. Yu S. Claude W. Ting-Shuang Y. . (2020). GetOrganelle: a fast and versatile toolkit for accurate de novo assembly of organelle genomes. Genome Biol. 21, 241. doi: 10.1186/s13059-020-02154-5 32912315 PMC7488116

[B9] Julio R. Albert F. Carlos S.-D. Sara G. R. Pablo L. Ramos-Onsins S. . (2017). DnaSP 6: DNA sequence polymorphism analysis of large data sets. Mol. Biol. Evol. 34, 3299–3021. doi: 10.1093/molbev/msx248 29029172

[B10] Kalyaanamoorthy S. Minh B. Wong T. Von Haeseler A. Jermiin L. (2017). ModelFinder: fast model selection for accurate phylogenetic estimates. Nat. Methods 14, 587–589. doi: 10.1038/nmeth.4285 28481363 PMC5453245

[B11] Katoh K. Standley D. (2013). MAFFT multiple sequence alignment software version 7: improvements in performance and usability. Mol. Biol. Evol. 30, 772–780. doi: 10.1093/molbev/mst010 23329690 PMC3603318

[B12] Kurtz S. Choudhuri J. Ohlebusch E. Schleiermacher C. Stoye J. Giegerich R. (2001). REPuter: the manifold applications of repeat analysis on a genomic scale. Nucleic Acids Res. 29, 4633–4642. doi: 10.1093/nar/29.22.4633 11713313 PMC92531

[B13] Li H. Jian J. Liu Y. Yang Z. (2026). Phylogenetic study and accurate identification of Tamarix species in China based on complete chloroplast genomes. BMC Plant Biol 26, 1119. doi: 10.1186/s12870-026-08959-z 42177439 PMC13326327

[B14] Li J. Wang S. Yu J. Wang L. Zhou S. (2013). A modified CTAB protocol for plant DNA extraction. Chin. Bull. Bot. 48, 72–78. doi: 10.3724/sp.j.1259.2013.00072

[B15] Li X. Yang Y. Henry R. Rossetto M. Wang Y. Chen S. (2015). Plant DNA barcoding: from gene to genome. Biol. Rev. 90, 157–166. doi: 10.1111/brv.12104 24666563

[B16] Liu Y. Chen K. Wang L. Yu X. Xu C. Suo Z. . (2025a). Assembly-free reads accurate identification (AFRAID) approach outperforms other methods of DNA barcoding in the walnut family (Juglandaceae). Plant Divers. 47, 115–126. doi: 10.1016/j.pld.2024.10.002 40041568 PMC11873577

[B17] Liu Y. Ding K. Liang L. Zhang Z. Chen K. Li H. (2024). Comparative study on chloroplast genome of Tamarix species. Ecol. Evol. 14, e70353. doi: 10.1002/ece3.70353 39360124 PMC11445282

[B18] Liu Y. Shen F. Wang L. Dou J. Dong T. Li M. . (2025b). Accelerating moss identification through the development of specific DNA barcodes based on the whole chloroplast genome. Mol. Ecol. Resour. 25, e70004. doi: 10.1111/1755-0998.70004 40590201

[B19] Liu Y. Xue N. Xu C. Wang Y. Huang X. Sun R. . (2025c). Species concept of golden flower camellias in China. BMC Plant Biol. 25, 1010. doi: 10.1186/s12870-025-07067-8 40751122 PMC12315351

[B20] Lubna Asaf S. Jan R. Asif S. Bilal S. Khan A. . (2024). Plastome diversity and evolution in mosses: Insights from structural characterization, comparative genomics, and phylogenetic analysis. Int. J. Biol. Macromol. 257, 128608. doi: 10.1016/j.ijbiomac.2023.128608 38065441

[B21] Makowska-Wąs J. Sobolewska D. Grabowska K. Wróbel-Biedrawa D. Podolak I. (2026). Genus Myricaria, the smaller sister of Tamarisks—Ornamental value, phytochemistry, biological activities and traditional uses. Life 16, 832. doi: 10.3390/life16050832 42195387 PMC13208939

[B22] Minh B. Schmidt H. Chernomor O. Schrempf D. Woodhams M. von Haeseler A. . (2020). IQ-TREE 2: new models and efficient methods for phylogenetic inference in the genomic era. Mol. Biol. Evol. 37, 1530–1534. doi: 10.1093/molbev/msaa015 32011700 PMC7182206

[B23] Mower J. Vickrey T. (2018). Chapter nine - structural diversity among plastid genomes of land plants. In Advances in Botanical Research, vol. 85, Eds. Chaw S.-M. Jansen R. (Lincoln: Academic Press), 263–292. doi: 10.1016/bs.abr.2017.11.013

[B24] Patel R. Jain M. (2012). NGS QC Toolkit: a toolkit for quality control of next generation sequencing data. PloS One 7, e30619. doi: 10.1371/journal.pone.0030619 22312429 PMC3270013

[B25] Ronquist F. Teslenko M. van der Mark P. Ayres D. Darling A. Höhna S. . (2012). MrBayes 3.2: efficient Bayesian phylogenetic inference and model choice across a large model space. Syst. Biol. 61, 539–542. doi: 10.1093/sysbio/sys029 22357727 PMC3329765

[B26] Shen X. Li Y. Jiang D. Liu Y. (2025). Creating an effective DNA identification system for discriminating cherries (Prunus subgenus Cerasus). BMC Plant Biol. 25, 475. doi: 10.1186/s12870-025-06328-w 40234750 PMC11998463

[B27] Sun J. Zhao D. Qiao P. Wang Y. Wu P. Wang K. . (2024). Phylogeny of genera in Maleae (Rosaceae) based on chloroplast genome analysis. Front. Plant Sci. 15, 1367645. doi: 10.3389/fpls.2024.1367645 38595768 PMC11002139

[B28] Talavera G. Castresana J. (2007). Improvement of phylogenies after removing divergent and ambiguously aligned blocks from protein sequence alignments. Syst. Biol. 56, 564–577. doi: 10.1080/10635150701472164 17654362

[B29] Tillich M. Lehwark P. Pellizzer T. Ulbricht-Jones E. Fischer A. Bock R. . (2017). GeSeq – versatile and accurate annotation of organelle genomes. Nucleic Acids Res. 45, W6–W11. doi: 10.1093/nar/gkx391 28486635 PMC5570176

[B30] Wang Y. Liu K. Li M. Bai Y. Zhang C. Yan B. (2025). Original species identification of Epimedii Folium (Epimedium) and their distributional responses to climate change. Sci. Traditional Chin. Med 3 (2), 178–185. doi: 10.1097/st9.0000000000000066 42348292

[B31] Wang Y. Liu Y. Liu S. Huang H. (2009). Molecular phylogeny of Myricaria (Tamaricaceae): implications for taxonomy and conservation in China. Bot. Stud. 50, 343–352. https://ejournal.sinica.edu.tw/bbas/content/2009/3/Bot503-10.pdf.

[B32] Wang Y. Liu H. Zhao D. Wang S. Wang J. Chi X. . (2026a). Assessment of suitable cultivation area for Paris polyphylla var. chinensis and var. yunnanensis under anthropogenic disturbance based on ensemble modeling and germplasm identification. BMC Plant Biol. 26, 241. doi: 10.1186/s12870-025-08010-7 41535733 PMC12874964

[B33] Wang Y. Yan P. Zhang Q. Wang J. Hao F. Xie W. . (2026b). Pan-chloroplast genome of Coptis species reveals genetic divergence and medicinal materials identification. Phytomedicine 159, 158485. doi: 10.1016/j.phymed.2026.158485 42378802

[B34] Yan X. Kan S. Wang M. Li Y. Tembrock L. He W. (2024). Genetic diversity and evolution of the plastome in allotetraploid cotton (Gossypium spp.). J. Syst. Evol. 62, 1118–1136. doi: 10.1111/jse.13070 40046247

[B35] Yu J. Xue J.-H. Zhou S.-L. (2011). New universal matK primers for DNA barcoding angiosperms. J. Syst. Evol. 49, 176–181. doi: 10.1111/j.1759-6831.2011.00134.x

[B36] Zhang M.-L. Meng H.-H. Zhang H.-X. Vyacheslav B. Sanderson S. (2014). Himalayan origin and evolution of Myricaria (Tamaricaeae) in the Neogene. PloS One 9, e97582. doi: 10.1371/journal.pone.0097582 24905234 PMC4048171

[B37] Zhang W. Sun Y. Liu J. Xu C. Zou X. Chen X. . (2021). DNA barcoding of Oryza: conventional, specific, and super barcodes. Plant Mol. Biol. 105, 215–228. doi: 10.22541/au.158592560.03447648 32880855 PMC7858216

[B38] Zhao D.-N. Ren Y. Zhang J.-Q. (2020). Conservation and innovation: plastome evolution during rapid radiation of Rhodiola on the Qinghai-Tibetan Plateau. Mol. Phylogenet. Evol. 144, 106713. doi: 10.1016/j.ympev.2019.106713 31863901

